# Studies of Immune Responses in *Candida vaginitis*

**DOI:** 10.3390/pathogens4040697

**Published:** 2015-10-10

**Authors:** Flavia De Bernardis, Silvia Arancia, Silvia Sandini, Sofia Graziani, Sandro Norelli

**Affiliations:** Department of Infectious, Parasitic and Immunomediated Diseases, Istituto Superiore di Sanità, Viale Regina Elena 299, 00161, Rome, Italy; E-Mails: silvia.arancia@iss.it (S.A.); silvia.sandini@iss.it (S.S.); sofia.graziani@iss.it (S.G.); sandro.norelli@iss.it (S.N.)

**Keywords:** *Candida vaginitis*, immune response, aspartyl proteinase, mucosal anti-*Candida* vaccine

## Abstract

The widespread occurrence of vaginal candidiasis and the development of resistance against anti-fungal agents has stimulated interest in understanding the pathogenesis of this disease. The aim of our work was to characterize, in an animal model of vaginal candidiasis, the mechanisms that play a role in the induction of mucosal immunity against *C. albicans* and the interaction between innate and adaptive immunity. Our studies evidenced the elicitation of cell-mediated immunity (CMIs) and antibody (Abs)-mediated immunity with a Th1 protective immunity. An immune response of this magnitude in the vagina was very encouraging to identify the proper targets for new strategies for vaccination or immunotherapy of vaginal candidiasis. Overall, our data provide clear evidence that it is possible to prevent *C. albicans* vaginal infection by active intravaginal immunization with aspartyl proteinase expressed as recombinant protein. This opens the way to a modality for anti-*Candida* protection at the mucosa. The recombinant protein Sap2 was assembled with virosomes, and a vaccine PEVION7 (PEV7) was obtained. The results have given evidence that the vaccine, constituted of virosomes and Secretory aspartyl proteinase 2 (Sap2) (PEV7), has an encouraging therapeutic potential for the treatment of recurrent vulvovaginal candidiasis.

## 1. Introduction

The majority of human infections by *Candida* occur at the mucosa [1,2]. Several epidemiological studies [3,4,5,6,7] have documented that vulvovaginal candidiasis is a widespread, common disease affecting up to 75% of healthy women, with some of them affected by recurrent, often intractable forms of the disease. Recurrent vulvovaginal candidiasis (RVVC) is a much more serious clinical condition due to the recurrences of symptoms (four or more episodes per year) and for its refractoriness to successful treatment. Long-term maintenance therapy with fluconazole may help lengthen the asymptomatic periods between recurrences, but does not provide a long-lasting cure [5]. Recent epidemiological investigations have suggested that the prevalence of RVVC may be higher than previously estimated and can be as high as 7%–8% of women who experience a first episode. In these cases, the quality of life is devastated, and the associated cost of medical visits is high. Anti-fungal therapy is highly effective for individual symptomatic attacks, but does not prevent recurrences. In fact, maintenance therapy with an efficacious anti-*Candida* drug lengthens the time to recurrence, but does not provide a long-term cure [5,6]. Furthermore, there is concern that repeated treatments might induce drug resistance, shift the spectrum of causative *Candida* species and result in an increased incidence of non-*C. albicans*, intrinsically-resistant species [6,7,8,9,10,11,12,13,14,15,16].

Moreover, in contrast to systemic candidiasis, relatively little is known about the role of mucosal immunity in protection against *Candida.* The widespread occurrence of mucosal candidiasis and the development of resistance against anti-fungal agents has stimulated interest in understanding the components of the host-fungus interaction at the mucosa and can result in the optimization of preventive and therapeutic antifungal strategies.

*C. albicans* is capable of colonizing and persisting on mucosa of the oral cavity and of the gastrointestinal and genitourinary tracts of healthy humans and also of stimulating mucosal responses. Odds [17] has suggested that 40%–50% of any given sample population temporarily or permanently carries this fungus in their gastrointestinal tract.

The virulence factors of *Candida* that play a role in mucosal infections are: adherence, dimorphisms with antigenic variations, enzyme production, especially proteinase secretion, and cell surface composition [18,19,20,21,22,23,24]. The formal demonstration of the role in infection has been obtained for some of these factors by the use of knockout mutants and reinsertion of relevant genes [19,20,21,25,26,27]. Adhesins play an important role in the pathogenesis of mucosal candidiasis by facilitating adherence to vaginal tissue [26,28,29,30]. Virulence expression is also promoted by the capacity of this fungus to form hyphae, *i.e.*, long, apically-growing threads endowed with a multiplicity of immune-evasion mechanisms and greatly favoring implantation on the mucosa [31,32,33]. There is clear evidence that the capacity of *C. albicans* to develop hyphae is required for vaginal infection [27,34,35,36]. Tissue sections of animal vaginas show that hyphae strongly adhere to the keratinized surface of the vaginal epithelium with some hyphal tips slightly infiltrating the subepithelial layer [36,37]. There is a clear demonstration that each deletion of relevant genes affecting hyphal transition determines the decrease or abolition of experimental pathogenicity [20,27].

Strains of *C. albicans* that lack the capacity to undergo the dimorphic transition are typically non-pathogenic [38,39]. Naglik and collaborators showed that the two forms of growth are discriminated by activation of distinct MAP kinase pathways [40].

Enzyme secretion, in particular aspartic proteinase (Sap), a family of at least 10 enzymes, plays a role in vaginal candidiasis. In fact, mutants of *C. albicans* with Sap1-3 knock-out genes do not cause vaginal infection in rats and lose the capacity to damage the reconstituted human vaginal epithelium, both pathogenic activities being regained following re-insertion of the relevant gene [25,41]. No such inference could be made with Sap4-6 KO mutants, even when the triple mutant was used [25].

In order to obtain possible insights into the host factors involved in the defense against vaginal candidiasis, we have long been employing a rat model of vaginal infection that has similarities to human disease, including the vaginal CD4/CD8 T-cell ratio [42,43]. In this model, an initial self-healing infection confers a high degree of protection against subsequent re-infection by *C. albicans* [42]. The protection is associated with the presence of protective antibodies against *Candida* constituents in the vaginal fluids and an increased number of activated lymphocytes in the vaginal mucosa [44,45]. The adoptive transfer of vaginal lymphocyte (VL) populations showed that distinct lymphocyte subsets participated in the adaptive anti-*Candida* immunity in the vagina and demonstrated not only that CD4^+^ T-cells were essential for protection, but also that other cellular types were probably involved [46,47]. Vaginal dendritic cells (VDCs) from infected rats induced the proliferation of T-cells and the release of high levels of IL-2, IFNγ and IL-6 and low levels of IL-10. The animals receiving VDCs from *C. albicans*-infected rats showed reduced (50%) *C. albicans* CFU counts [48].

Moreover, the specific objectives of our works were to identify the proper targets for new strategies for vaccination or immunotherapy of vaginal candidiasis.

The data reported in this review are a summary of our studies on the mechanisms induced in the vagina during *Candida* infection and of our research to identify specific *Candida* molecules potentially useful for vaccination or immunotherapy of vaginal candidiasis.

More detailed information about the epidemiology, diagnosis, current treatments of the infection and recent studies for the development of protective vaccine are included in excellent reviews already published on this subject [5,12,49,50,51,52,53,54,55].

## 2. Summary of Our Studies to Identify the Proper Targets for New Strategies for Vaccination or Immunotherapy of Vaginal Candidiasis

The evidence of an immune response in the vagina was very encouraging to identify the proper targets for new strategies for vaccination or immunotherapy of vaginal candidiasis. Active intravaginal immunization with native mannoprotein (MP) or secretory aspartyl proteinase (Sap) conferred an elevated degree of antibody-mediated protection against vaginal infection by *C. albicans* [25,42,44,45]. Furthermore, we have evidenced that intranasal and intravaginal immunizations with MP or Sap and CT, as mucosal adjuvant, were equally effective at inducing specific antibody response in the vagina and conferring a high degree of protection against vaginal infection by *C. albicans* [56]. In the context of generating a candidate vaccine against mucosal candidiasis, the availability of a recombinant product would greatly assist overcoming the well-known difficulties in obtaining, purifying and standardizing a native antigen. We were working with two recombinant proteins: an aspartyl-proteinase Sap2 and a protein of 65 kDa that we call MP65, which are important immune-dominant antigens and virulence factors of *C. albicans* acting in mucosal infections [35,57].

Overall, the results of our studies evidenced that intravaginal immunization with recombinant MP65 mannoprotein (r MP65 r or secretory aspartyl proteinase (r Sap2) conferred protection against vaginal candidiasis. Below, we reported examples of experiments, already performed, of the kinetics of vaginal infection in rats immunized intravaginally with the recombinant protein: r MP65 or r Sap2 [35,56,57].

**Figure 1:**
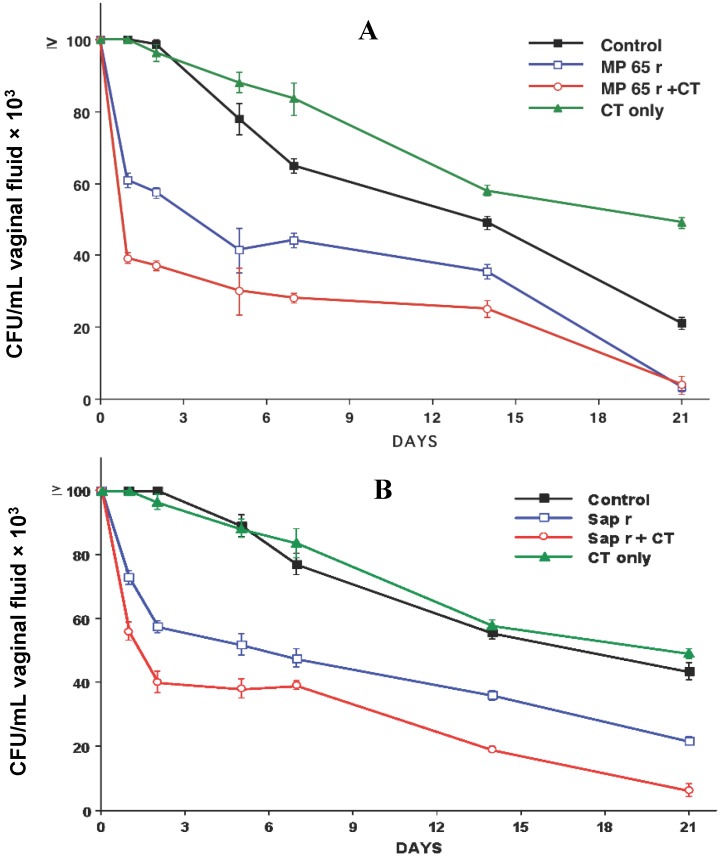
(**A**) Vaginal colonization of *C. albicans* in rats immunized with recombinant MP65; (**B**) vaginal colonization of *C. albicans* in rats immunized with recombinant Sap2.

As shown in Figure 1A (which refers to one typical experiment out of four experiments performed with similar results), the rats immunized intravaginally with MP were characterized by early clearance of the fungal cells from the vagina, as compared to rats given the adjuvant or saline only, as demonstrated by nearly a 50% reduction of vaginal *Candida* counts by the first 48 h after challenge. This early, two-day clearance rate was significantly more pronounced in the animals immunized with the antigen plus adjuvant cholera toxin (CT), an effect that persisted for at least two weeks after challenge [35,56].

As shown in Figure 1B (which refers to one typical experiment out of four experiments that had similar results), immunization with the recombinant Sap2 resulted in statistically-significant acceleration of fungal clearance from the vagina in the first week of infection, both when the animals were only immunized with the antigen and, more significantly, when CT was co-administered with Sap2. The effect on fungal clearance somewhat faded away in the second and third week of infection. However, on Day 21, all CT plus Sap2-immunized animals were cleared of the infection, whereas all controls were still infected. No effect on clearance was shown by administration of CT only [56,57].

Then, we focused on Sap2 as a target, as it is the most characterized virulence trait of the fungus: a family of ten genes. It has a clear role in mucosal infection by *C. albicans*. It was overexpressed in the vagina and oral cavity, but not systemically. The Sap2-deleted mutant becomes avirulent, while that that is Sap2-reconstructed regains total virulence. Inhibition of Sap2 expression by pepstatin A exerts a marked therapeutic effect *in vivo*. Furthermore, Sap2 is involved in adhesion.

Our data provided clear evidence that it is possible to prevent *C. albicans* vaginal infection by active intravaginal immunization with aspartyl proteinase expressed as recombinant protein. This opened the way to a modality for anti-*Candida* protection in the mucosa [57]. 

## 3. Summary of Our Studies for the Development of a Protective Vaccine for *C. albicans* Vaginal Infections

A few years ago, we started a collaboration with Pevion biotech, a Swiss Company that produces a vaccine consisting of virosomes. The virosomes were assembled with the recombinant protein Sap2, and a vaccine PEVION7 (PEV7) was obtained, which had Sap2 antigen presented on the surface of virosomes.

We examined whether the virosomal vaccine PEV7 conferred protection to rats experimentally challenged with *C. albicans*. For this purpose, rats were immunized with three intravaginal administrations of the vaccine and were challenged with *Candida* cells one month after the last immunization. 

Immunization with the virosomal vaccine conferred a substantial protection from *C. albicans* challenge, as evidenced by the accelerated clearance of the *Candida* cells from the vagina and resolution of the infection at least one week before infection in controls: non-immunized rats or animals receiving only empty virosomes.

In the vaginal fluids of rats vaccinated with virosomes and Sap2, anti-proteinase antibodies (IgG and IgA) have been detected.

The results have given evidence that the vaccine constituted of virosomal and Sap2 (PEV7) has an encouraging therapeutic potential for the treatment of recurrent vulvovaginal candidiasis [58].

After these preclinical studies, a phase I clinical trial was designed and performed by Pevion to assess the safety and immunogenicity of the PEV7 vaccine in healthy volunteers. Half of the subjects received intramuscular injections, while the other half will receive capsules, administered intravaginally. In total, the study enrolled 48 healthy women of childbearing age. The study demonstrated the generation of specific and functional B-cell memory in 100% of the vaccinated women. Half of the volunteers vaccinated with the intramuscular low dose of PEV7 received a single booster immunization 14 months after the primary vaccination course. All routes of vaccination showed a rapid and specific response, either in serum and/or in cervicovaginal secretion. An immune response of this magnitude in the cervicovaginal compartment was very encouraging with regards to the therapeutic potential of the vaccine [59]. 

NovaDigm Therapeutics Inc. is a company developing innovative vaccines for fungal and bacterial infections. Its products include NovaDigmVaccine-3 (NDV-3), a vaccine contains the Als3 antigen, which facilitates *Candida* adherence to and invasion of human endothelial cells. This vaccine is currently in a phase 1b/2a clinical study for the prevention of recurrent vulvovaginal candidiasis [60]. Moreover, as described on the website (www.novadigm.net), the company has acquired the rights, in four separate transactions, to three well-studied *Candida* vaccine antigens. The three antigens acquired are hyphally-regulated protein 1 (Hyr1), secreted aspartyl proteinase 2 (Sap2) and a β-mannan conjugate. Hyr1 was licensed from the Los Angeles BioMedical Research Institute at Harbor-UCLA Medical Center. The use of recombinant Hyr1 as a protective antigen was discovered by NovaDigm’s founding scientists, led by John E. Edwards, Jr., MD, Chair of the Division of Infectious Diseases [61,62]. Rights to Sap2 were acquired from Pevion, a Swiss biotech company, and Istituto Superiore di Sanità (ISS) in Rome, Italy.

Rights to the β-mannan trisaccharide conjugate were acquired from three leading academic researchers: David Bundle, PhD, Professor of Chemistry at the University of Alberta, Edmont AB, Canada; Jim E. Cutler, PhD, Professor (retired), Pediatrics and Microbiology, Immunology and Parasitology, School of Medicine; Louisiana State University, New Orleans, Louisiane, USA, and Mark Nitz, PhD, Professor of Chemistry, University of Toronto, Toronto, ON, Canada [63].

Hyr1 is a component of the *Candida* cell wall that inhibits the innate immune system’s ability to kill *Candida*. In preclinical studies, an Hyr1 vaccine conferred protection from systemic candidiasis in murine models by producing antibodies that reversed the inhibition of the immune system. Sap2 degrades essential components of the immune response and contributes to inflammation. A phase 1 study of a Sap2-based vaccine conducted by Pevion demonstrated favorable safety and immunogenicity, inducing the production of anti-Sap2 antibodies, which are thought to neutralize Sap2’s involvement in immune evasion and to inhibit inflammatory responses. β-mannan is a key outer cell wall component of *Candida*, which may be involved in adhesion to host cells. Vaccines based on the conjugation of β-mannan to a protein carrier have demonstrated protection against both systemic and vaginal *Candida* infections in numerous preclinical studies.

The aim of the NovaDigm Company is to produce a multivalent vaccine that can induce an immune response against multiple virulence traits of *Candida* and can enhance the probability of success against *C. albicans* mucosal infections [60].

## 4. Conclusions

The high incidence of RVVC and the difficulties controlling its occurrence with conventional anti-mycotic therapy constitute a strong medical need for the development of immunological treatments adding to, if not replacing, the current antifungal treatment. The use of exogenous cytokines, antibodies and immune-modulators is of potential interest [64,65], but the development of a safe and efficacious anti-*Candida* vaccine may be a better approach. Recently, several research teams developed anti-*Candida* vaccination [57,58,66,67]. Two of these vaccines have passed phase 1 clinical trials for safety and immunogenicity, and one of them has entered a phase 2 clinical trial. Both vaccines evidenced protection in rat and mouse models of vaginal infection by *C. albicans*, although with a slightly different mechanism of immunological protection [49,50,51].

Other anti-*Candida* vaccines, from attenuated strains of *C. albicans* [52,68] to a number of glycoconjugate of cell wall polysaccharides [69,70], have also been shown to be immunogenic and protective in experimental animal models, although these have not yet been entered into clinical trials in humans. However, *Candida* species contain a range of factors that facilitate tissue invasion by enabling the fungus to evade, modulate or exacerbate the host’s immune system. Thus, a multi-antigen vaccine approach could significantly weaken the ability of *Candida* to escape from the body’s immune system and provide a more effective vaccine. The combination of antigens that are related to *C. albicans* virulence factors may induce additive or synergistic immune responses, broadening the spectrum of protective antibodies and reducing the probability of fungal immune evasion. In addition, combined vaccines are likely to be more effective in protecting against the different site- and tissue-specific *C. albicans* infections, which have different mechanisms of pathogenicity [71].

It is hoped that gynecologists will be offered a safe and protective anti-*Candida* vaccine in the not too distant future. In fact, a mucosal vaccine would improve the quality of life of the high number of women affected with RVVC.

## References

[1] Nucci M., Marr K.A. (2005). Emerging Fungal Diseases. Clin. Inf. Dis..

[2] Pfaller M.A., Diekema D.J. (2007). Epidemiology of invasive candidiasis: A persistent public health problem. Clin. Microbiol. Rev..

[3] Sobel J.D. (2002). Pathogenesis of Recurrent Vulvovaginal Candidiasis. Curr. Infect. Dis. Rep..

[4] Nyirjesy P., Sobel J.D. (2003). Vuvovaginal candidiasis. Obstet. Gynecol. Clin. N. Am..

[5] Sobel J.D., Wiesenfield H.C., Martens M., Danna P., Hooton T.M., Rompalo A., Sperling M., Livengood C., Horowitz B., von Thron J. (2004). Maintenance therapy for recurrent vulvovaginal candidiasis. N. Engl. J. Med..

[6] Sobel J.D. (2006). Management of recurrent vulvovaginal candidiasis: Unresolved issues. Curr. Infect. Dis. Rep..

[7] Foxman B., Muraglia R., Dietz J.P., Sobel J.D., Wagner J. (2013). Prevalence of recurrent vulvovaginal candidiasis in 5 European countries and the United States: Results from an internet panel survey. J. Low. Genit. Tract Dis..

[8] Bauters T.G., Dhont M.A., Temmerman M.I., Nelis H.J. (2002). Prevalence of vulvovaginal candidiasis and susceptibility to fluconazole in women. Am. J. Obstet. Gynecol..

[9] Cernicka J., Subik J. (2006). Resistance mechanisms in fluconazole resistant *Candida albicans* isolates from vaginal candidiasis. Int. J. Antimicrob. Agents.

[10] Jackson S.T., Mullings A.M., Rainford L., Miller A. (2005). The epidemiology of mycotic vulvovaginitis and the use of antifungal agents in suspected mycotic vulvovaginitis and its implications for clinical practice. West Indian Med. J..

[11] Richter S.S., Galask R.P., Messer S.A., Hollis R.J., Diekema D.J., Pfaller M.A. (2005). Antifungal susceptibilities of *Candida* species causing vulvovaginitis and epidemiology of recurrent cases. J. Clin. Microbiol..

[12] Ringdahl E.N. (2006). Recurrent vulvovaginal candidiasis. Mol. Med..

[13] Ventolini G., Baggish M.S., Walsh P.M. (2006). Vulvovaginal candidiasis from non-*albicans* species: retrospective study of recurrence rate after fluconazole therapy. J. Reprod. Med..

[14] Shahid Z., Sobel J.D. (2009). Reduced fluconazole susceptibility of *Candida albicans* isolates in women with recurrent vulvovaginal candidiasis: Effects of long-term fluconazole therapy. Diagn. Microbiol. Infect. Dis..

[15] Marchaim D., Lemanek L., Bheemreddy S., Kaye K.S., Sobel J.D. (2012). Fluconazole-resistant *Candida albicans* vulvovaginitis. Obstet. Gynecol..

[16] Kennedy M.A., Sobel J.D. (2010). Vulvovaginal Candidiasis Caused by Non-*albicans Candida* Species: New Insights. Curr. Infect. Dis. Rep..

[17] Odds F.C. (1988). Chronic mucocutaneous candidosis. Candida and Candidosis.

[18] De Bernardis F., Cassone A., Sturtevant J., Calderone R. (1995). Expression of *Candida albicans* SAP1 and SAP2 in experimental vaginitis. Infect. Immun..

[19] De Bernardis F., Sullivan P.A., Cassone A. (2001). Aspartyl proteinases of *Candida albicans* and their role in pathogenicity. Med. Mycol..

[20] Calderone R., Fonzi W. (2001). Virulence factors of *Candida albicans*. Trends Microbiol..

[21] Hube B. (2004). From commensal to pathogen: stage and tissue specific gene expression of *Candida albicans*. Curr. Opin. Microbiol..

[22] Naglik J.R., Challacombe S.J., Hube B. (2003). *Candida albicans* secreted aspartyl proteinases in virulence and pathogenesis. Microbiol. Mol. Biol. Rev..

[23] Thewes S., Kretschmar M., Park H., Schaller M., Filler S.G., Hube B. (2007). *In vivo* and *ex vivo* comparative transcriptional profiling of invasive and non-invasive *Candida albicans* isolates identifies genes associated with tissue invasion. Mol. Microbiol..

[24] Mayer F.L., Wilson D., Hube B. (2013). *Candida albicans* pathogenicity mechanisms. Virulence.

[25] De Bernardis F., Arancia S., Morelli L., Hube B., Sanglard D., Schafer W., Cassone A. (1999). Evidence that members of the secretory aspartyl proteinases gene family (SAP), in particular SAP2, are virulence factors for *Candida vaginitis*. J. Infect. Dis..

[26] Hoyer L.L. (2001). The ALS gene family of *Candida albicans*. Trends Microbiol..

[27] Kumamoto C.A., Vinces M.D. (2005). Contribution of hyphae and hypha-co-regulated genes to *Candida albicans* virulence. Cell. Microbiol..

[28] Sundstrom P. (2002). Adhesion in *Candida* spp.. Cell. Microbiol..

[29] Spellberg B.J., Ibrahim A.S., Avanesian V., Fu Y., Myers C., Phan Q.T., Filler S.G., Yeaman M.R., Edwards J.E. (2006). Efficacy of the anti-*Candida* rAls3p-N or Als1p-N vaccines against disseminated and mucosal candidiasis. J. Infect. Dis..

[30] Zordan R., Cormack B., Calderone R.A., Clancy C.J. (2012). Adhesins on Opportunistic Fungal Pathogens. Candida and Candidiasis.

[31] Sudbery P., Gow N., Berman J. (2004). The distinct morphogenic states of *Candida albicans*. Trends Microbiol..

[32] Sudbery P.E. (2011). Growth of *Candida albicans* hyphae. Nat. Rev. Microbiol..

[33] Jacobsen I.D., Wilson D., Wächtler B., Brunke S., Naglik J.R., Hube B. (2012). *Candida albicans* dimorphism as a therapeutic target. Expert Rev. Anti Infect. Ther..

[34] Saville S.P., Lazell A.L., Monteagudo C., Lopez-Ribot J.L. (2003). Engineered control of cell morphology *in vivo* reveals distinct roles for yeast and filamentous forms of *Candida albicans* during infection. Eukaryot. Cell..

[35] Sandini S., la Valle R., de Bernardis F., Macri C., Cassone A. (2007). The 65-kilodalton mannoprotein gene of *Candida albicans* encodes a putative glucanase adhesin required for hyphal morphogenesis and experimental pathogenicity. Cell. Microbiol..

[36] De Bernardis F., Molinari A., Boccanera M., Stringaro A., Robert R., Senet J.M., Arancia G., Cassone A. (1994). Modulation of cell surface-associated mannoprotein antigen expression in experimental candidal vaginitis. Infect. Immun..

[37] De Bernardis F., Liu H., O’Mahony R., la Valle R., Bartollino S., Sandini S., Grant S., Brewis N., Tomlinson I., Basset R.C. (2007). Human domain antibodies against virulence traits of *Candida albicans* inhibit fungus adherence to vaginal epithelium and protect against experimental vaginal candidiasis. J. Infect. Dis..

[38] Lo H., Kholer J.R., di Domenico B., Loebenberg D., Cacciapuoti A., Fink G.R. (1997). Nonfilamentous *C. albicans* mutants are avirulent. Cell.

[39] Peters B.M., Palmer G.E., Nash A.K., Lilly E.A., Fidel P.L., Noverr M.C. (2014). Fungal morphogenetic pathways are required for the hallmark inflammatory response during *Candida albicans* vaginitis. Infect. Immun..

[40] Moyes D.L., Murciano C., Runglall M., Islam A., Thavaraj S., Naglik J.R. (2011). *Candida albicans* yeast and hyphae are discriminated by MAPK signaling in vaginal epithelial cells. PLoS ONE.

[41] Schaller M., Borelli C., Korting H.C., Hube B. (2005). Hydrolytic enzymes as virulence factors of *Candida albicans*. Mycoses.

[42] Cassone A., Boccanera M., Adriani D., Santoni G., de Bernardis F. (1995). Rat clearing a vagina infection by *Candida albicans* acquired specific antibody-mediated resistance to vaginal reinfection. Infect. Immun..

[43] Elitsur Y., Jackman S., Neace C., Keerthy S., Liu X., Dosescu J., Moshier J.A. (1998). Gen. Diagn. Pathol..

[44] De Bernardis F., Boccanera M., Adriani D., Spreghini E., Santoni G., Cassone A. (1997). Protective role of antimannan and anti-aspartyl proteinase antibodies in an experimental model of *Candida albicans* vaginitis in rats. Infect. Immun..

[45] De Bernardis F., Santoni G., Boccanera M., Spreghini E., Adriani D., Morelli L., Cassone A. (2000). Local anticandidal immune responses in a rat model of vaginal infection by and protection, *Candida albicans*. Infect. Immun..

[46] Santoni G., Boccanera M., Adriani D., Lucciarini R., Amantini C., Morrone S., Cassone A., de Bernardis F. (2002). Immune cell-mediated protection against vaginal candidiasis: Evidence for a mayor role of vaginal CD^4+^ T cells and possible participation of other local lymphocyte effectors. Infect. Immun..

[47] De Bernardis F., Santoni G., Boccanera M., Lucciarini R., Arancia S., Sandini S., Amantini C., Cassone A. (2010). Protection against rat vaginal Candidiasis by adoptive transfer of vaginal B lymphocytes. FEMS Yeast Res..

[48] De Bernardis F., Lucciarini R., Boccanera M., Amantini C., Arancia S., Morrone S., Mosca M., Cassone A., Santoni G. (2006). Phenotypic and functional characterization of vaginal dendritic cells in a rat model of *Candida albicans* vaginitis. Infect. Immun..

[49] Cassone A. (2008). Fungal vaccines: Real progress from real challenges. Lancet Infect. Dis..

[50] Cassone A., Casadevall A. (2012). Recent progress in vaccines against fungal diseases. Curr. Opin. Microbiol..

[51] Cassone A. (2013). Development of vaccines for *Candida albicans*: Fighting a skilled transformer. Nat. Rev. Microbiol..

[52] Edwards J.E. (2012). Fungal cell wall vaccines: an update. J. Med. Microbiol..

[53] Iannitti R.G., Carvalho A., Romani L. (2012). From memory to antifungal vaccine design. Trends Immunol..

[54] Moragues M.D., Rementeria A., Sevilla M.J., Eraso E., Quindos G. (2014). *Candida* antigens and immune responses: Implications for a vaccine. Expert Rev. Vaccines.

[55] Peters B.M., Yano J., Noverr M.C., Fidel P.L. (2014). *Candida vaginitis*: When Opportunism Knocks, the Host Responds. PLoS Pathog..

[56] De Bernardis F., Boccanera M., Adriani D., Girolamo A., Cassone A. (2002). Intravaginal and intranasal immunizations are equally effective in inducing vaginal antibodies and conferring protection against vaginal candidiasis. Infect. Immun..

[57] Sandini S., la Valle R., Deaglio S., Malavasi F., Cassone A., de Bernardis F. (2011). A highly immunogenic recombinant and truncated protein of the secreted aspartic proteases family (rSap2t) of *Candida albicans* as a mucosal anticandidal vaccine. FEMS Immunol. Med. Microbiol..

[58] De Bernardis F., Amacker M., Arancia S., Sandini S., Gremion C., Zurbriggen R., Moser C., Cassone A. (2012). A virosomal vaccine against candidal vaginitis: Immunogenicity, efficacy and safety profile in animal models. Vaccine.

[59] PEV7 Clinical Trial. http://www.clinicaltrials.gov/ct2/show/NCT01067131.

[60] ClinicalTrials.gov Identifier: NCT01926028. NCT01926028.

[61] Luo G., Ibrahim A.S., Spellberg B., Nobile C.J., Mitchell A.P., Fu Y. (2010). *Candida albicans* Hyr1p Confers Resistance to Neutrophil Killing and Is a Potential Vaccine Target. J. Infect. Dis..

[62] Luo G., Ibrahim A.S., French S.W., Edwards J.E., Fu Y. (2011). Active and Passive Immunization with rHyr1p-N Protects Mice against Hematogenously Disseminated Candidiasis. PLoS ONE.

[63] Nitz M., Ling C., Otter A., Cutler J.E., Bundle D.R. (2002). The Unique Solution Structure and Immunochemistry of the *Candida albicans* β-1,2-Mannopyranan Cell Wall Antigens. J. Biol. Chem..

[64] Magliani W., Conti S., de Bernardis F., Cassone A., Polonelli L. (2002). New immunotherapeutic strategies to control vaginal candidiasis. Trends Mol. Med..

[65] Ostrowski-Zeichner L., Casadevall A., Galgiani J.N., Odds F.C., Rex J.H. (2010). An insight into the antifungal pipeline: selected new molecules and beyond. Nat. Rev. Drug Discov..

[66] Cutler J.E., Deepe G.S., Klein D. (2007). Advances in combating fungal diseases: Vaccines on the threshold. Nat. Rev. Microbiol..

[67] Schmidt C.S., White C.J., Ibrahim A.S., Filler S.G., Fu Y., Yeaman M.R., Edwards J.E., Hennessey J.P. (2012). NDV-3, a recombinant alum-adjuvanted vaccine for *Candida* and *Staphylococcus aureus*, is safe and immunogenic in healthy adults. Vaccine.

[68] Saville S.P., Lazzell A.J., Chaturvedi A., Monteagudo C., Lopez-Ribot J.L. (2009). Efficacy of a genetically engineered *Candida albicans* tet-NRG1strain as an experimental live attenuated vaccine against hemathogeneously disseminated candidiasis. Clin. Vaccine Immunol..

[69] Xin H., Dziadek S., Bundle D.R., Cutler J.E. (2008). Synthetic glycopeptide vaccines combining beta-mannan and peptide epitopes induce protection against candidiasis. Proc. Natl. Acad. Sci. USA.

[70] Bromuro C., Romano M., Chiani P., Berti F., Tontini M., Proietti D., Mori E., Torosantucci A., Costantino P., Rappuoli R. (2010). Beta-glucan-CRM197 conjugates as candidates antifungal vaccines. Vaccine.

[71] Cassone A., Cauda R. (2012). *Candida* and candidiasis in HIV-infected subjects. Where commensalism, opportunistic behavior and frank pathogenicity lose their borders. AIDS.

